# Anti-diabetic potential of S*apium ellipticum* (Hochst) Pax leaf extract in Streptozotocin(STZ)-induced diabetic Wistar rats

**DOI:** 10.1186/s12906-017-2013-8

**Published:** 2017-12-08

**Authors:** Osasenaga Macdonald Ighodaro, Oluseyi Adeboye Akinloye

**Affiliations:** 1 Department of Biochemistry, Faculty of Sciences, Lead City University, Ibadan, Nigeria; 2 Department of Biochemistry, College of Biosciences, Federal University of Agriculture, Abeokuta (FUNAAB), Abeokuta, Nigeria

**Keywords:** *Sapium Ellipticum*, Diabetes, Streptozotocin, Insulin, Glycogen

## Abstract

**Backgound:**

Ethnobatanical survey associates *Sapium ellipticum* (SE) with antidiabetic usage among other medicinal functions in different parts of Africa. More importantly, previous studies on the plant extract in our laboratory showed that SE has significant effects on the activities of carbohydrate metabolizing enzymes such as glucokinase, glucose-6-phosphatase, α-amylase and α-glucosidase. In view of these, the anti-diabetic potential of the plant leaf extract in streptozotocin (STZ)-induced diabetes rat model (Wistar strain) was examined.

**Methods:**

Diabetes was induced in experimental animals via single intraperitoneal dose (55 mg/kg BW) of freshly prepared STZ. SE was evaluated at 400 and 800 mg kg^−1^ of body weight (BW), against metformin (12 mgkg^−1^ BW). Treatments were done orally (p.o), twice daily at 8 h interval for a period of 21 days.

**Results:**

SE significantly reduced fasting blood glucose (FBG) level by 46.5 and 44.4% (400 and 800 mg dosage respectively) compared to initial diabetic values. However, the effects were significantly lower than 72.6% glucose reduction produced by metformin. Hepatic and skeletal muscle glycogens were observed to increase by 27.06 and 12.55% respectively in SE-treated rats (800 mg dosage) compared to their corresponding values in diabetic control animals. Plasma and pancreatic insulin contents were also improved (31.77 and 52.34% respectively) by SE administration. The histopathological examination of the pancreas indicates beta cells regeneration in the treated animals, particularly in diabetic rats treated with 800 mg dosage of the extract compared to the diabetic control animals and metformin group. The presence of phenolic compounds namely amentoflavone, lupeol and luteolin-7-O-glucoside in SE as characterized and reported in our previous study is likely responsibly for the antidiabetic effects of the plant extract noted in the present study.

**Conclusion:**

The outcome of this study provides scientific basis in support of the medicinal relevance of SE and lend credence to its utilization in folk medicine for the treatment of diabetes and other oxidative stress-related ailments.

## Background

The incidence of diabetes is alarming and has exceeded epidemic proportion particularly among the upper age cadre. Its prevalence as at 2010 is estimated to be 5.2% of the global population, affecting over 285 million people worldwide. The number of people affected is still on the rise and estimated to escalate to 438 million by 2030 [1]. The therapeutic options available for diabetes are insulin and four classes of oral anti-diabetic drugs that stimulate pancreatic insulin secretion and insulin action. However, there is yet no effective cure for diabetes and the available drugs and insulin currently used in managing the disease are associated with multiple undesirable side effects [2, 3]. This limitation has necessitated the need for natural materials with hypoglycemic properties and their employment in the management of diabetes [4, 5].

Over the years, the use of plants and plant products in ethno medicine for the treatment of various diseases has become popular and widely acceptable all over the world. World Health Organization (WHO) estimates that about 80% of the people in developing countries depend mainly on traditional medicine for their primary health care and 85% of such traditional medicine involves the use of plant extract [5–7]. The reason for this lies in the fact that plants generally contain variety of chemical compounds with significant biological functions. Most of these bioactive substances elicit long-term health benefits in humans when consumed, and can be used to effectively treat human diseases.

*S. ellipticum* belongs to the family *Euphorbiaceae* and is commonly referred to as jumping seed tree. It is widely distributed in eastern and tropical Africa. In southwest part of Nigeria, particularly among the Ilorin indigenes, the plant is popularly known as *aloko-agbo.* According to Burkill [8, 9], a number of therapeutic usage is associated with the plant in folklore medicine. For instance, preparation of dried leaves of *S.ellipticum* is applied to wounds in Tanzania; the leaf preparation is also used for sore-eyes and abdominal swelling. In Central Africa, the decoction of the stem bark is used for scurvy and stomatitis, as purgative in Congo and for treatment of eczema. Interestingly, it is believed to be a cure for stammering in Zaire. In Tanganyika, a leaf-preparation is used to relieve pains in the head, chest, shoulders and back. A root-concoction is prepared as a fomentation in East Africa for enlarged spleen in babies and is taken by adults for malaria. Cytotoxicity screening of selected Nigerian plants used in traditional cancer treatment on HT29 (colon cancer) and MCF-7 (breast cancer) cell lines indicated that *Sapium ellipticum* leaf extract expressed the highest cytotoxic activity among other plants with anticancer potential [10]. The antioxidant properties of the stem bark extract of the plant has been reported by Adesegun et al. [11].

Previous studies on *Sapium ellipticum* extract in our laboratory showed that it has significant effects on carbohydrate metabolizing enzymes such as α-amylase and α-glucosidase in vitro [12], glucokinase and glucose-6-phosphatase in vivo [13]. Moreover, HPLC-MS analysis of its fractions also revealed among other bioactive compounds the presence of amentoflavone [14] which has been associated with anti-diabetic function [15]*.*

Hence, this study sought to evaluate the anti-diabetic potential of *Sapium ellipticum* ethanol leaf extract in streptozotocin-induced diabetic rats, vis-à-vis fasting blood glucose (FBG), glycogen and insulin concentrations as well as pancreatic beta cell architecture. Since animals, precisely mammals and humans share striking similarity in body metabolism, the outcome of this study may have relevance and application to humans.

## Methods

### Collection of *Sapium ellipticum* and preparation of leaf extracts

Fresh *Sapium ellipcitum* leaves were harvested in the month December 2012 from a forest in a suburb of Ibadan, southwest of Nigeria. The harvested leaves were taxonomically authenticated by a curator botanist (Mr. T.K. Odewo) at the Lagos University Herbarium (LUH), Nigeria, were a specimen was deposited to obtain a voucher specimen number LUH 5423. The plant material was freed of extraneous materials; air dried at room temperature and was milled into a fine powder with a milling machine. SE extract was prepared by macerating 50 g of the dried powdery sample in 250 mL of the extracting solvent (absolute ethanol) at room temperature. The mixture was allowed to stand for 72 h and stirred intermittently to facilitate extraction. After which it was filtered using a muslin cloth of mesh size, 42. The resulting volume on sieving was reduced with a rotary evaporator. Final solvent elimination and drying was done using a water bath at 40 °C. The crude extracts were stored in sterile screwed (air-tight) bottles and aliquots were taken when required.

### Collection and management of animals

Male adult albino rats of the Wistar strain (150–220 g body weight) were used for the study. They were obtained from the animal breeding unit of Institute for Advance Medical Research and Training (IMRAT), at the University College Hospital (UCH), Ibadan, Nigeria. All procedures for maintenance and sacrifice (care and use) of animals were carried out according to the criteria outlined by the National Academy of Science published by the National Institute of Health [16]. This was approved by the Ethical Committee of the College of Bioscience, Federal University of Agriculture, Abeokuta. The animals were handled humanely, kept in plastic suspended cages, placed in a well ventilated and hygienic rat house under suitable conditions of temperature and humidity. They were provided rat pellets (Ladokun feeds) and served water ad libitum and subjected to natural photoperiod of 12 h light and 12 h dark cycle. The animals were allowed 2 weeks of acclimatization, body weights and blood glucose levels (baseline measurement) were estimated prior to the commencement of all experiments in this study.

### Acute effect of SE extract on fasting blood glucose (FBG) in normoglycaemic rats

Thirty male adult rats were randomly assigned to 5 groups (*n* = 6) and were fasted overnight for 12 h. Using aseptic precautions, blood glucose levels were determined in the animals prior to any form of treatment as described in 2.7.1. Thereafter, group 1 animals were treated with corn oil; vehicle for the extract (0.5 mL). Groups 2, 3, 4 and 5 received 200, 400, 800 and 1000 mg of SE per kilogram body weight of rats respectively. Blood glucose concentration was monitored for over a period of 6 h at 2 h interval (post treatment with corn oil or SE). The animals were not allowed access to laboratory chow and water ad libitum during the experiment.

### Effect of SE extract on oral glucose tolerance (OGT) in normoglycaemic rats

Based on the outcome of the preceding experiment, 800 mg of SE per kg BW of rat which produced the maximum reduction of fasting blood glucose level was used in this evaluation. Twelve rats were fasted for 12 h and assigned randomly to 2 groups, each containing 6 rats. The initial fasting blood glucose levels were estimated prior to treatments. The control animals received corn oil (0.5 mL, p.o) while the experimental animals were administered SE (800 mg/kgBW, p.o). 30 min, after the administrations, all rats in both groups were orally loaded with 10 mL 50% (*w*/*v*) of glucose solution per kilogram body weight. Blood glucose concentration was immediately determined in rats and monitored at 1 hour interval up to 4 h after glucose challenge.

### Induction of diabetes mellitus with streptozotocin in experimental rats

Single intraperitoneal (i.p) dose (55 mgKg^−1^BW) of freshly prepared streptozotocin (STZ) was administered to a batch of normoglycaemic rats starved for 16 h. Forty-eight hours after STZ injection, acutecheck active glucometer with disposable test strips was used to determine the fasting blood glucose level of rats. Animals with 200 mg/dL and above were considered to be diabetic and used for the study. In order to reduce mortality rate which usually occurs in diabetic animals as a result of hypoglycemic shock, the animals were placed on 5% glucose solution for the first 24 h of the study.

### Anti-diabetic effects of SE extract

Eight normoglycemic animals constituted a control group (group 1). Thirty-two diabetic Wistar rats were randomly assigned to four groups (groups 2, 3, 4 and 5) containing eight animals each. Group 1 animals were administered olive oil (0.5 mL) and served as normal control. Group 2 animals were left untreated and served as diabetic control. Groups 3 and 4 were respectively treated with 400 and 800 of SE kg^−1^BW and the last group was treated with metformin (12 mgkg^−1^ BW), a reference anti-diabetic drug. All treatments were done orally (p.o), twice daily at 8 h interval for a period of 21 days. All groups of animals were allowed equal access to normal laboratory chow and water ad libitum*.*

### Blood glucose determination

Blood glucose was monitored at interval of 7 days. The acucheck active glucometer with disposable test strips containing a chemically treated test spot was used to measure the amount of blood glucose. Blood samples were collected from the tails of the animals. The tail was first wiped with surgical spirit and then nibbed with a pair of sharp scissors. Test Strip was fully inserted into the meter before applying a drop of blood to fully cover the test area inside the grey target. The principle of the test is based on a glucose oxidase/peroxidase reaction, which is specific for ß-D-glucose. The test area of the strip is designed in such a way that when a drop of blood is placed on the top surface, color change occurs which is determined by the glucometer and this is proportional to the concentration of glucose in the blood sample. After collection of blood, the nibbled side of the tail was rubbed with cotton wool soaked in absolute ethanol to protect the animal from infection and to arrest further bleeding.

### Muscle and liver glycogen determination

Glycogen contents in hepatocytes and myocytes were determined by the method of Seifter with slight modifications as reported in Methods in Enzymology Vol. 111 [17]. By this method, the tissues were digested in hot concentrated KOH, glycogen was precipitated with ethanol, washed and re-precipitated, and finally glycogen concentration was determined with a spectrophotometer at a wavelength of 620 nm, using anthrone as the reagent.

### Isolation of organs and histopathological examination of pancreas

After the last glucose level determination, the animals were fasted overnight and weighed and then sacrificed by cervical dislocation. Organs including the heart, liver, kidney, spleen and pancreas were harvested rinsed with 1.15% KCl, blotted and weighed. A thin section of the pancreas was prepared for histopathological examination.

### Estimation of hepatic and muscle glycogen contents in rats

Glycogen contents in hepatocytes and myocytes were determined by the method of Seifter with slight modifications as reported in Methods in Enzymology Vol. 111 [17].

### Estimation of serum and pancreatic insulin level in rats

Serum and pancreatic insulin were measured by an enzyme-linked immunosorbent assay (ELISA) procedure using rat insulin ELISA kit (USA).

### Statistical analysis of data

Data analysis was performed using statistical software, Graphpad Prism, version 6.4. The statistical significance of difference between groups was analyzed using the one-way analysis of variance (ANOVA) followed by independent-sample t test. The level of significance was set at *p* < 0.05. The results are presented as the mean ± SD or mean ± SEM (*n* = 6).

## Results

### Acute effect of SE extract on FBGL in normoglycaemic rats

**Fig. 1:**
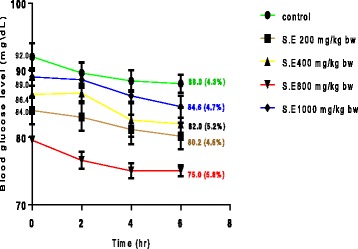
Acute effect of SE on fasting blood glucose level (FGBL) in normoglycemic rats. Values are expressed as mean of six rats. Figures in parentheses are mean percentage reduction in fasting blood glucose level (FGBL)

Figure 1 shows the acute effect of graded doses of SE on fasting blood glucose level (FGBL) of rats. After 6 hours of administration, the extract at all evaluated doses caused slight reduction (ranging from 4.5 to 5.8%) in FGBL. The highest reduction (5.8%) was observed in rats treated with 800 mg/kg BW which was not significant when compared with the level of reduction recorded in control animals (4.3%) and other experimental groups (4.5, 4.7 and 5.2%).

### Effect of SE on oral glucose tolerance (OGT) in normoglycaemic rats

**Fig. 2:**
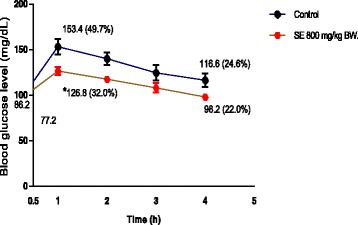
Oral glucose tolerance effect of SE in normoglycemic rats. Values are expressed as mean of six rats. Figures in parentheses are mean percentage reduction in fasting blood glucose level (FGBL). * = significant when compared to control

There was no significant difference in the oral glucose tolerance capacity of control rats and those pre-treated with SE extract. Nonetheless, the extract apparently suppressed the degree of hyperglycaemia induced by glucose loading in rats by 17.0% when the increase in blood glucose (32%) is compared to the 49.7% level of hyperglycaemia noted in the control animals as depicted in Fig. 2.

### 3. 3. Anti-diabetic effects of SE extract

**Fig. 3:**
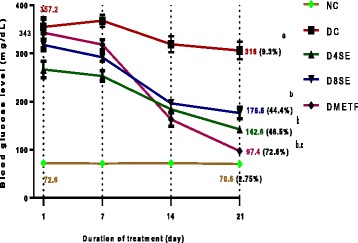
Curative effect of SE and METF on Blood glucose concentration of STZ-Treated rats. Values are expressed as mean ± SEM of 8 rats. Figures in parentheses represent mean percentage reduction in fasting blood glucose. NC = Normal control, DC = Diabetic control, D4SE = Diabetic animals treated with SE (400 mg\kg BW), D8SE = Diabetic animals treated with SE (800 mg\kg BW), DMETF = Diabetic animals treated with metformin (12 mg\kg BW). a = significant when compared to NC, b = significant when compared to DC, c = significant when compared D4SE/D8SE, d = significant when compared to DMETF

Figure 3 represents the effects of treatments of diabetic rats with SE extract (400 and 800 mg/kg BW) and METF (12 mg/kg BW). Particularly from the 14th day of treatment, blood glucose level was observed to decline gradually in all groups. This trend continues during the study and the last glucose estimation which was on the 21st day showed that the control rats recorded the least reduction in blood glucose (2.75%) whereas rats treated with SE extract significantly reduced FBGL by 46.5 and 44.4% (at 400 and 800 mg dosage respectively) compared to their initial diabetic values. However, the effects were significantly lower than the 72.6% glucose reduction produced by METF. The hypoglycemic effects of both SE extract and METF were dependent on the duration of administration; as lesser FBG reductions were noticed on the third and seventh day of treatment.

**Fig. 4:**
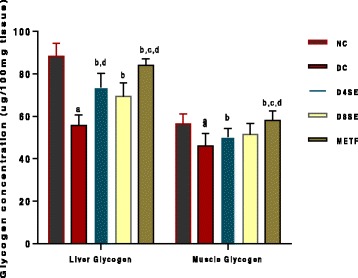
Effects of SE and METF on liver and muscle Glycogen concentration in STZ-Treated rats. Values are expressed as mean ± SEM of 8 rats. NC = Normal control, DC = Diabetic control, D4SE = Diabetic animals treated with SE (400 mg\kg BW), D8SE = Diabetic animals treated with SE (800 mg\kg BW), DMETF = Diabetic animals treated with metformin (12 mg\kg BW). a = significant when compared to NC, b = significant when compared to DC, c = significant when compared D4SE d = significant when compared to D8SE, f = significant when compared to DMETF

Figure 4 represents the effects of SE and METF treatments on hepatic and muscle glycogen contents in STZ-diabetic rats. Liver and muscle glycogen were significantly lowered in diabetic control animals relatively to normal control rats, with more severity noted in the liver (36.94%) relative to 18.51% decrease observed in the muscle. The extract at the administered doses improved both hepatic and muscle glycogen contents in rats with 400 mg dosage producing a significantly (*p* <0.05) greater improvement (32.37 and 9.41%) compared to 800 mg dosage (27.06 and 12.25%). The enhancing effects of both doses were significantly lower than that of METF in both tissues.

**Fig. 5:**
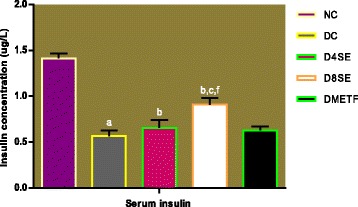
Effect of SE and METF on serum insulin concentration in rats. Values are expressed as mean ± SEM of 8 rats. NC = Normal control, DC = Diabetic control, D4SE = Diabetic animals treated with SE (400 mg\kg BW), D8SE = Diabetic animals treated with SE (800 mg\kg BW), DMETF = Diabetic animals treated with metformin (12 mg\kg BW). a = significant when compared to NC, b = significant when compared to DC, c = significant when compared D4SE d = significant when compared to D8SE, f = significant when compared to DMETF

**Fig. 6:**
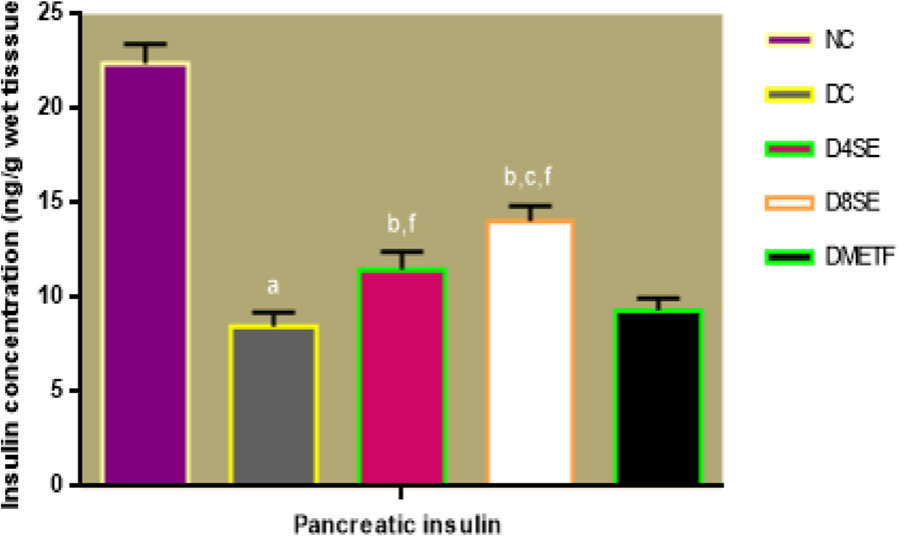
Effect of SE and METF on serum and pancreatic insulin concentration in rats. Values are expressed as mean ± SEM of 8 rats. NC = Normal control, DC = Diabetic control, D4SE = Diabetic animals treated with SE (400 mg\kg BW), D8SE = Diabetic animals treated with SE (800 mg\kg BW), DMETF = Diabetic animals treated with metformin (12 mg\kg BW). a = significant when compared to NC, b = significant when compared to DC, c = significant when compared D4SE d = significant when compared to D8SE, f = significant when compared to DMETF

Figures 5 and 6 respectively describe the effects of SE extract and METF on serum and pancreatic insulin in STZ-diabetic rats. The levels of insulin in the serum and pancreas were significantly decreased in diabetic control rats relative to normal control animals. The extract at the administered doses (400 and 800 mg/kg BW) significantly raised the level of insulin in both serum and pancreas relative to diabetic control rats. Compared to SE, treatment of diabetic rats with METF failed to significantly restore the level of insulin in both tissues.

### Effects of SE extract on the histological architecture of the pancreas

**Fig. 7:**
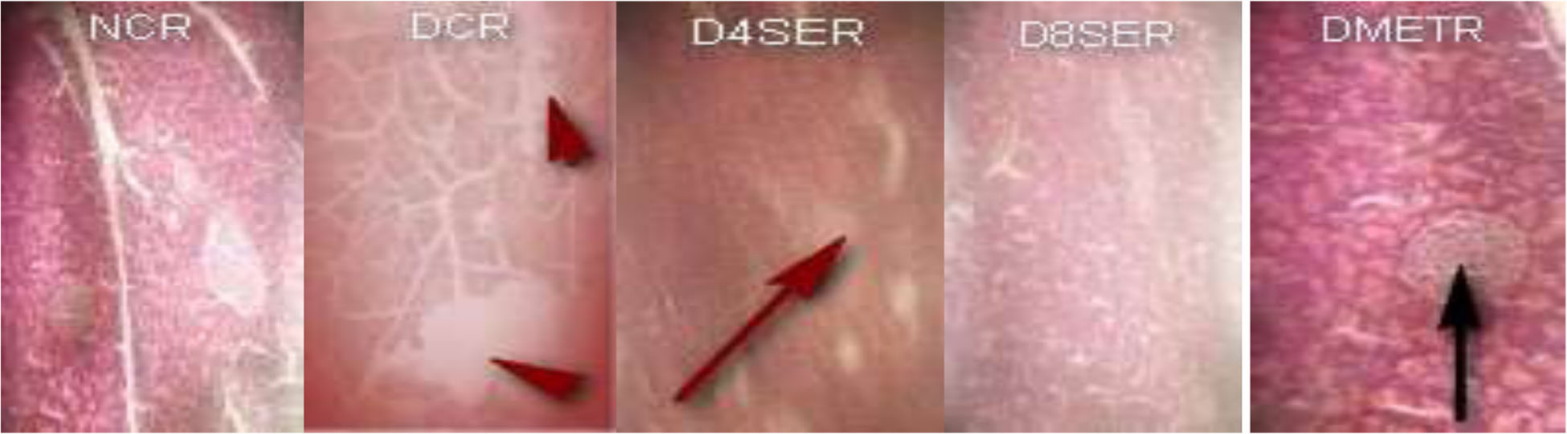
Effects of SE extract on the histological architecture of the pancreas. **NCR:** Normal control rat showing normal pancreatic architecture with intact exocrine tissue and islet cells surrounded by delicate capsule. **DCR:** Diabetic control rat with exocrine tissue showing massive degeneration and endocrine tissue exhibiting hyperplasia reminiscent of hypoglycaemia. **D4SER:** Diabetic rat treated with 400 mg of SE per kg BW (p.o) showing mild atrophy of the pancreatic cell compared to DCR. **D8SER:** Diabetic rat treated with 800 mg of SE per kg BW (p.o) showing pancreatic cells regeneration to near normal. **DMETR:** Diabetic rats treated with 12 mg of Metformin per kg BW (p.o) showing pancreatic histology similar to that of control animal but dented with mild pancreatic cells congestion or occlusion

The photomicrographs of thin sections of pancreas excised from normal, untreated diabetic and treated diabetic rats are shown in Fig**.** 7.

## Discussion

N-methylnitrocarbamoyl-D-glucosamine popularly referred to as streptozotocin (STZ) is a well known and established diabetic agent used as a screening model for evaluating the antidiabetic potential or capacity of compounds in a wide range of animals [18, 19]. The compound (STZ) is a potent methylating agent for DNA and acts as nitric oxide donor in pancreatic cells. Pancreatic cells are particularly sensitive to damage by nitric oxide (via inhibition of aconitase activity) and free radicals because of their low levels of free radical scavenging enzymes [20]. Basically, STZ induces diabetes by destroying the insulin secreting pancreatic β-cells, resulting in a decrease in endogenous insulin release.

In accordance, findings from the current investigation showed that STZ administration (i.p) at 55 mg/kg BW effectively induced diabetes mellitus in physiologically normal rats as reflected by hyperglycemia, pancreatic beta cells degeneration, decreased insulin and glycogen levels in diabetic control animals. In contrast, SE leaf extract at the administered doses (400 and 800 mg/kg BW) significantly lowered STZ-induced hyperglycemia, improved insulin and glycogen contents in extract-treated rats compared to diabetic control animals.

Improvement in oral glucose tolerance capacity is one of the several mechanisms by which diabetes is controlled and managed. In this study, pre-treatment of rats with SE leaf extract at 800 mg/kg BW slightly improve oral glucose tolerance by 2.6%. In addition, the extract appeared to have significantly suppressed the degree of hyperglycemia induced by glucose loading by 17.7% in treated rats compared to control animals. This connotes that SE extract may be capable of providing some degree of delay in the onset of diabetes. Improved glucose tolerance is associated with the availability and functionality of plasma insulin [21]. This is evident in the present study in which treatment of rats with the extract significantly improved the level of insulin both in the serum and pancreas. This is may have arguably play a critical role in the hypoglycemic ability exhibited by the extract in lowering STZ-induced hyperglycemia in the treated animals relative to the diabetic control animals. The observed increase in both serum and pancreatic insulin concentrations is obviously connected to the ability of the extract to protect the animals against STZ- induced beta cell degeneration, and consequent activation of insulin production and release. This view is substantiated by the histopathological evaluation outcome of the pancreas of rats from the different groups of animals which showed that SE treated diabetic animals (particularly at 800 mg/kg BW) demonstrated relatively better histological architecture (beta cells regeneration), as well as higher levels of serum and pancreatic insulin compared to other groups of animals.

The liver in conjunction with extra hepatic tissues plays a significant role in glucose homeostasis. It absorbs about 35% of the postprandial glucose and effectively converts the molecules to glycogen through the process of glycogenesis for storage when there is sufficient insulin in circulation [22]. In response to lack of glucose availability in cells during diabetes (insufficiency or insensitivity of insulin), the organ accommodates the breakdown of glycogen to release glucose through glycogenolysis. Therefore, the observed reduction in glycogen level in the diabetic animals is likely a direct consequence of hydrolysis of the molecule in the liver as a responsive mechanism to maintain appropriate glycemic condition or indirect effect of insulin insufficiency or insensitivity.

In the same vein, increase in insulin production contributed to the significant increase in hepatic and skeletal muscle glycogen contents observed in the extract-treated diabetic rats compared to the diabetic control animals in which glycogen content was significantly decreased in both tissues. El-Shenawy and Abdel-Nabi [23] communicated similar opinion in their report on insulin treatment of diabetic animals. Besides, the natural ability of the liver to synthesize glycogen is severely impaired in hyperglycemic condition. This is because the main glycogenic enzyme (glycogen synthase) which catalyze the polymerization of glucose to form glycogen is down regulated while glycogen phosphorylase, an enzyme which catalyzes glycogen breakdown is up regulated in diabetes [24]. More so, the activities of these enzymes are insulin-dependent [25, 26]. While the latter is activated by increase serum insulin, the former is deactivated by the same condition, resulting in defective glycogen storage and excessive glycogen hydrolysis in the diabetics. However, it is noteworthy, that though METF produced significantly lower restorative effects on both serum and pancreatic insulin level, the hepatic and muscle glycogen contents of METF-treated diabetic rats were significantly higher than those estimated in their SE-treated counterparts. This is a bit contradictory in light of the direct correlation that exists between insulin level and glycogen content. What probably accounts for this is that METF may have possibly mimicked insulin (insulinotropic properties) and directly activated glycogen synthase or suppressed the activity of glycogen phosphorylase. Since these enzymes were not assayed in this work, it is difficult to make definite conclusion.

The antidiabetic potential shown by SE extract in this study is suggestive of the presence of one or more bioactive compounds with antidiabetic functions in the plant. We had previously identified some bioactive compounds namely amentoflavone, lupeol, luteolin-7-O-glucoside and alpha tocopherol in SE through HPLC-MS analysis [14]. Interestingly some of these compounds (amentoflavone and lupeol) have been associated with antidiabetic functions in other plants in previous studies by other researchers. For instance, Patil et al. [15] identified amentoflavone as the antidiabetic principle in *Biophytum sensitivum.* Lupeol has been reported to be one of the antidiabetic compounds in *Coccinia indica* [27] and two other *coccinia* species [28]. This is the first scientific report on the antidiabetic activity of *Sapium ellipticum* and the presence of these compounds in the plant is arguably connected with this medicinal property.

## Conclusion

Overall, findings of the current study provide scientific basis in support of the antidiabetic potentials of SE leaf extract and lend substantial credence to the use of the plant extract in folk medicine for the treatment of patients suffering from diabetes and other oxidative stress related disorders. Further studies are in progress in our laboratory to assess the antidiabetic effects of the isolated compounds individually and collectively.

## Abbreviations

BW: Body weight
DM: Diabetes mellitus
FBGL: Fasting blood glucose level
I.P: Intraperitoneal
METF: Metformin
P.O: Per ox
SE: Sapium ellipticum
STZ: Streptozotocin
